# Periodontal soft tissue increase induced by periodontally accelerated osteogenic orthodontics surgery

**DOI:** 10.1186/s12903-022-02566-8

**Published:** 2022-11-16

**Authors:** Ye Han, Lili Miao, Jian Liu, Xiao Xu, Zhaoguo Yue, Min Xu, Chang Shu, Li Xu, Jianxia Hou

**Affiliations:** Department of Periodontology, Material Technology of Stomatology, Beijing Key Laboratory of Digital Stomatology, Research Center of Engineering and Technology for Computerized Dentistry Ministry of Health, NMPA Key Laboratory for Dental Materials, Peking University School and Hospital of Stomatology, National Center of Stomatology, National Clinical Research Center for Oral Diseases, National Engineering Laboratory for Digital, Beijing, 100081 China

**Keywords:** Periodontally accelerated osteogenic orthodontics, Gingival thickness, Keratinized gingiva width, Soft tissue increase, Digital measurement

## Abstract

**Objectives:**

To quantitatively assess periodontal soft tissue changes, including gingival thickness and keratinized gingiva width after periodontally accelerated osteogenic orthodontics (PAOO) surgery by digital measurements.

**Methods:**

This study enrolled 15 maxillaries with 89 anterior teeth and 16 mandibles with 94 anterior teeth from Chinese adult patients with skeletal Angle Class III malocclusion for whom PAOO surgery was proposed during orthodontic treatment. Intraoral scanning and cone beam computed tomography (CBCT) examinations were performed before PAOO surgery and 6 months after the surgery. Keratinized gingiva width was measured on the digital model acquired by intraoral scanning. The gingival thickness was measured using a digital three-dimensional (3D) model based on the combination of digital intraoral scanning and CBCT data.

**Results:**

The mean gingival thickness before surgery was 0.91 ± 0.32 mm and 1.21 ± 0.38 mm at 6-month after PAOO. Patients showed periodontal soft tissue increase with a mean gingival tissue gain of 0.30 ± 0.33 mm. At 1 mm, 2 and 3 mm apical to cemento-enamel junction (CEJ) levels, the gingival thickness increase of the mandible was higher than that of the maxilla (0.38 ± 0.30 mm vs. 0.24 ± 0.31 mm, 0.43 ± 0.35 mm vs. 0.26 ± 0.41 mm, 0.36 ± 0.27 vs. 0.25 ± 0.32 mm, respectively, all *P* < 0.05). Moreover, the sites of gingival thickness ≤ 1 mm before surgery showed more tissue gain than the sites > 1 mm (0.36 ± 0.32 mm vs. 0.18 ± 0.31 mm, *P* < 0.001). The mean keratinized gingiva width at T0 was 3.88 ± 1.22 mm, and increased 1.05 ± 1.24 mm 6 months after PAOO surgery. Moreover, a digital 3D model for gingival thickness measurement based on the combination of digital intraoral scanning and CBCT displayed high reliability and accuracy with an intra-class correlation coefficient (ICC) of 0.897.

**Conclusion:**

PAOO could improve an insufficient quantity of periodontal soft and hard tissues in patients with skeletal Angle Class III malocclusion, including the gingival thickness and keratinized gingiva width. A digital 3D model based on the combination of digital intraoral scanning and CBCT data could provide a new digital measurement of gingival thickness with high accuracy and reliability.

**Supplementary Information:**

The online version contains supplementary material available at 10.1186/s12903-022-02566-8.

## Introduction

In recent years, periodontally accelerated osteogenic orthodontics (PAOO) has been widely used to achieve more stable periodontal soft and hard tissues, a shorter treatment time, and a reduced amount of apical root resorption during orthodontic treatment [1]. The surgery procedures of PAOO combine full-thickness flap elevation, alveolar corticotomy, bone grafting and guided tissue regeneration, and the application of orthodontic forces. Our previous studies proved that PAOO is effective and safe for the gingiva and alveolar bone [2–4]. The preliminary results further showed PAOO could improve insufficient periodontal soft and hard tissues, because a mean gain of keratinized gingiva thickness of 0.5 mm and of labial bone thickness gain of 0.7 mm was achieved [3].

However, few studies have focused on the changes of gingival thickness induced by PAOO [5]. Various methods have been attempted to measure the gingival thickness, including transgingival probing, evaluation of probe transparency through the tissues, ultrasonographic devices and more recently, cone beam computed tomography (CBCT), however, no one technique has been widely used because of invasiveness or the complexity of application [6]. Emerging intraoral scanning technology allows easier and noninvasive capture of digital data of the soft tissue. Additionally, dedicated three-dimensional (3D) analysis software provides an accurate measurement [7]. Therefore, we proposed a quantitative measurement method of gingival thickness based on the combination of intraoral scanning and CBCT data.

Our previous studies found a gain of keratinized gingiva after PAOO [3], which was in agreement with another study [8], and a gain of supracrestal gingival thickness six-month after PAOO surgery [4]. However, the investigation of the changes of soft tissue, particular gingival thickness induced by PAOO still remains limited. Therefore, this study was designed to quantitatively analyze the changes of periodontal soft tissue following PAOO by digital measurement based on the combination of intraoral scanning and CBCT data.

## Materials and methods

This study was approved by the Research Ethics Committee of Peking University Health Science Center (approval no. PKUSSIRB-201,735,074), registered in the Chinese Clinical Trial Registry (no. ChiCTR1900021778, 09/03/2019) and conducted in accordance with the Helsinki Declaration of 1975, as revised in 2013. All protocols were performed in accordance with approved guidelines and regulations, and written informed consent was obtained from all participants.

### Patient selection

Subjects with skeletal Angle Class III malocclusion who were advised by orthodontists, periodontists, and maxilla-facial surgeons to undergo PAOO due to the thinness of their labial alveolar bone before orthodontic decompensation were enrolled in the present study.

Inclusion criteria were: (1) aged 18–35 years; (2) skeletal Angle Class III malocclusion with the requirement for orthodontic and orthognathic treatment; (3) periodontal health, defined as probing depth ≤ 3 mm and bleeding on probing ≤ 10%; (4) labial alveolar bone thickness at the anterior teeth (maxillary and/or mandibular) *<* 0.5 mm, as demonstrated by CBCT; (5) no smoking history; and (6) systemic health. Exclusion criteria were: (1) pregnancy or lactation; (2) uncontrolled periodontal infection; (3) history of periodontal surgical treatment on the anterior teeth; (4) systemic disease or use of medication known to affect periodontal status; and (5) cleft lip/palate or maxillofacial abnormality.

### Surgical and orthodontic procedures

Following study enrollment, routine initial periodontal therapy (oral hygiene instructions, prophylaxis, and scaling and root planing as needed) was performed. Periodontal surgeries were performed by the same experienced periodontist(Li Xu)using surgical loupes (Zeiss 53 to 20, 3.5X; Carl Zeiss Meditec, Jena, Germany). Following local anesthesia administration, crevicular incisions were made on the buccal aspect (first premolar to first premolar) using microsurgical instruments. Full-thickness flaps were elevated and a Piezo-Surgical knife (OT7S-4; PiezoSurgery, Mectron, Italy) was used to create vertical interproximal alveolar decortication below the alveolar crest to a depth of 2 to 3 mm. Bone derivative material (Bio-Oss, 0.5 g; Geistlich, Switzerland) was grafted onto the labial aspect of the decorticated anterior cortical bone, into dehiscence and fenestrations, in the coronal-apical direction. Bioabsorbable collagen membrane (Bio-Gide, 25 mm × 25 mm; Geistlich, Switzerland) was adapted to completely cover the graft site. Flaps were coronally repositioned with single-sling suture and interrupted interdental sutures using non-absorbable 5.0 Prolene (Ethicon US, CA). Amoxicillin (500 mg/thrice a day for 7 days) and 0.12% chlorhexidine (10ml/twice a day for 14 days) were recommended after surgery. Ibuprofen was used at 0.3 g every 12 h within 3 days after surgery when patient felt intense pain. Sutures were removed 2 weeks post-operatively. Orthodontic forces were applied 2 weeks after the periodontal surgery.

### Intraoral scanning and CBCT examinations

All examinations were performed before PAOO surgery (T0) and 6 months after surgery (T1). An experienced operator used an intraoral scanner (3Shape Trios, 3Shape, Denmark) to obtain the digital impression at T0 and T1. CBCT scans were taken using a NewTom VG device (Aperio Services, Italy) at T0 and T1. The Standard Tessellation Language (STL) files of digital impression data and Digital Imaging and Communications in Medicine (DICOM) files of alveolar bone 3D reconstruction data were acquired.

### Gingival thickness and keratinized gingiva width measurement

**Fig. 1:**
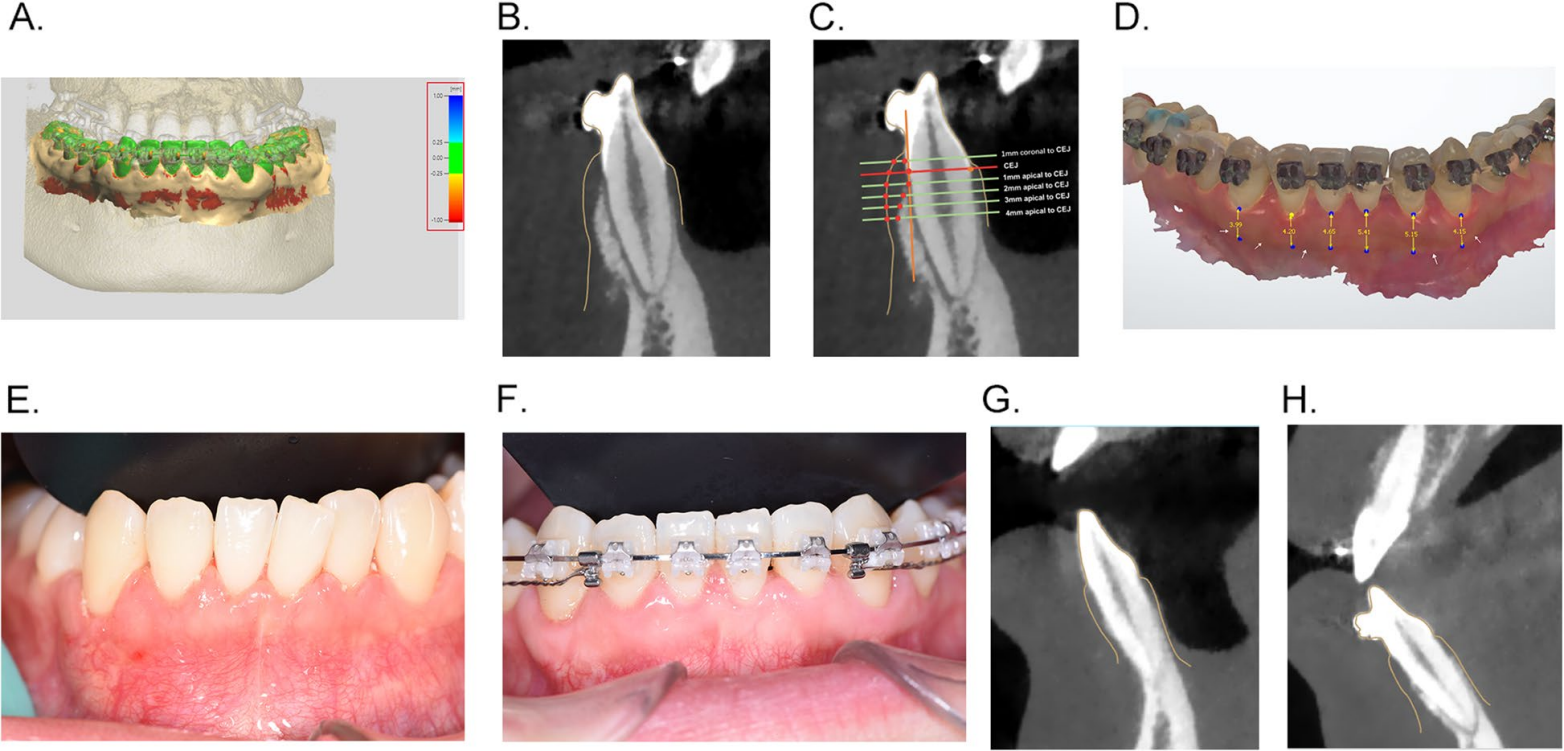
**A** The registration of intraoral scanning and CBCT data; **B** The model of the combination of intraoral scanning and CBCT data for gingival thickness measurement; **C** The measurement levels of gingival thickness on the combination model; **D** The measurement of keratinized gingiva width on the intraoral scanning model; **E** The representative patient intraoral photograph at T0; **F** The representative patient intraoral photograph at T1; **G** The representative patient combination model at T0; **H** The representative patient combination model at T1

These data were imported to the 3Shape Design Studio software (Denmark) for gingival thickness measurement. Automatic registration algorithms were applied for the accurate registration using the corresponding anatomical surfaces as a common structure in both intraoral scanning and CBCT images. The color bar on the Fig. 1 A displays the distances of the two models after registration and is used as the reference for deviation. The green area represents an accurate automatic registration with system error < 250 μm, while red or blue represents a relatively large deviation. In the case of a visible deviation between the images, manual registration is needed to adjust by manually moving intraoral scanning and CBCT images in 3D space.

Following registration, a new 3D model of the combination of intraoral scanning and CBCT images were generated. The gingival thickness was recorded as the distance between the mucosa surface and the bone surface at the planes made perpendicular to the long axis of the teeth (Fig. 1B). The measurement levels were at 1 mm coronal to cemento-enamel junction (CEJ) level, CEJ level, 1 mm, 2 mm, 3 mm, 4 mm to CEJ (Fig. 1 C).

The keratinized gingiva width was measured on the color digitalization model acquired by intraoral scanning. The demarcation (arrows on Fig. 1D) between the attached gingiva and the darker alveolar mucosa was the mucogingival junction. The keratinized gingiva width was defined as the distance between the gingival margin and the mucogingival junction at the midfacial aspect of each anterior tooth and the mucogingival junction.

All measurements were performed by two independent examiners blinded to the data information. The consistency of each of the two examiners was evaluated by the intra-class correlation coefficient (ICC).

### Statistical analysis

Variables are presented as mean ± standard deviation (SD; normal distribution). ICC was used to evaluate the inter-examiner reliabilities of gingival thickness measurements. On the basis of the study by Landis and Koch (1997), the ICC scale was interpreted as follows: poor to fair (≤ 0.4), moderate (0.41–0.60), excellent (0.61–0.80), and almost perfect (0.81–1) [9]. Comparisons of gingival thickness and keratinized gingiva width between T0 and T1 were performed using paired samples t-test. All statistical analyses were performed using SPSS v20.0 software (IBM Corp., Armonk, NY, USA). A two-tailed *p*-value < 0.05 was considered to be statistically significant.

## Results

### Patient population and surgical procedures

Twenty-four patients (nine men and 15 women, aged 18 to 30 years) were included in the study. Fifteen maxillaries with 89 anterior teeth and 16 mandibles with 94 anterior teeth were included for measurements. The intraoral images showed the effect of periodontal soft tissue increase 6 months after PAOO surgery (Fig. 1E,F), and Fig. 1G,H showed the gain of gingival thickness on the measurement model of the same patient.

### Accuracy and reliability of digital measurement model for gingival thickness measurement

Figure 1B shows a 3D model of the combination of intraoral scanning and CBCT images. The corresponding tooth surfaces of intraoral scanning and CBCT were used for registration, which was the green color in Fig. 1 A. This green color represents areas where no volumetric change occurred, which confirmed the accuracy of the combination model (Fig. 1 A).

The gingival thickness was measured as the distance between from the surfaces of mucosa as detected by intraoral scanning to the bone surface as detected by CBCT. Inter-examiner reliability of this method was examined by ICC. An ICC of 0.897, proved the reliability of this digital measurement model.

### The changes of gingival thickness

A gain of gingival thickness was observed 6 months after PAOO surgery at all measurement levels (Table 1). For all measured sites, the mean gingival thickness before surgery was 0.91 ± 0.32 mm and the average gingival thickness was 1.21 ± 0.38 mm at 6 months after PAOO, thus there was a mean gain of gingival thickness of 0.30 ± 0.33 mm (*p* < 0.001). The gingival thickness before surgery was 0.86 ± 0.32 mm, 1.17 ± 0.33 mm, 1.00 ± 0.29 mm, 0.91 ± 0.31 mm, 0.83 ± 0.26 mm, and 0.75 ± 0.22 mm at 1 mm coronal to CEJ level, CEJ level, 1 mm, 2 mm, 3 mm, 4 mm to CEJ level respectively. The gingiva tended to become thinner the more apical it was to the CEJ. Six months after PAOO, the gingival thickness increased significantly by 0.19 ± 0.27 mm, 0.31 ± 0.35 mm, 0.28 ± 0.33 mm, 0.33 ± 0.40 mm, 0.33 ± 0.29 mm, and 0.30 ± 0.33 mm at each of the respective levels. The increase of gingival thickness at 1 mm coronal to CEJ was significantly less than for the other measurement levels, while there was no statistic difference among the other measurement levels.

**Table 1:** The measurements of gingiva thickness (mm) before PAOO and 6-month after surgery

Measurement levels	T0	T1	Difference(T1-T0)	*P*
1 mm coronal to CEJ	0.86 ± 0.32	1.04 ± 0.39	0.19 ± 0.27	< 0.001***
CEJ	1.17 ± 0.33	1.48 ± 0.46	0.31 ± 0.35	< 0.001***
1 mm apical to CEJ	1.00 ± 0.29	1.28 ± 0.34	0.28 ± 0.33	< 0.001***
2 mm apical to CEJ	0.91 ± 0.31	1.24 ± 0.35	0.33 ± 0.40	< 0.001***
3 mm apical to CEJ	0.83 ± 0.26	1.12 ± 0.30	0.30 ± 0.30	< 0.001***
4 mm apical to CEJ	0.75 ± 0.22	1.09 ± 0.31	0.33 ± 0.29	< 0.001***
all sites	0.91 ± 0.32	1.21 ± 0.38	0.30 ± 0.33	< 0.001***

Data are presented as mean ± SD/N

*T0* before periodontally accelerated osteogenic orthodontics surgery, *T1* 6 months after surgery, *CEJ* cemento-enamal junction

^***^P < 0.001

The analysis of the gingival thickness increase differences between maxilla and mandible showed that there were no significant differences at all the sites (maxilla vs. mandible: 0.30 ± 0.35 mm vs. 0.32 ± 0.30 mm), while at 1 mm, 2 and 3 mm apical to CEJ level, the increase of the mandible was higher than the maxilla (*P* < 0.05, Table 2).

**Table 2:** The differences of gingiva thickness increase (mm) before PAOO and 6-month after surgery at different jaw

	maxilla			mandible			*P*
Measurement levels	T0	T1	Difference(T1-T0)	T0	T1	Difference(T1-T0)	
1 mm coronal to CEJ	0.97 ± 0.27	1.20 ± 0.34	0.23 ± 0.31***	0.71 ± 0.32	0.83 ± 0.34	0.13 ± 0.19***	0.089
CEJ	1.29 ± 0.33	1.66 ± 0.44	0.36 ± 0.38***	0.93 ± 0.21	1.18 ± 0.36	0.24 ± 0.30***	0.105
1 mm apical to CEJ	1.09 ± 0.29	1.31 ± 0.38	0.24 ± 0.31***	0.88 ± 0.24	1.26 ± 0.28	0.38 ± 0.30***	0.016*
2 mm apical to CEJ	0.97 ± 0.34	1.23 ± 0.37	0.26 ± 0.41***	0.82 ± 0.25	1.25 ± 0.33	0.43 ± 0.35***	0.016*
3 mm apical to CEJ	0.87 ± 0.30	1.12 ± 0.31	0.25 ± 0.32***	0.76 ± 0.18	1.12 ± 0.28	0.36 ± 0.27***	0.045*
4 mm apical to CEJ	0.83 ± 0.21	1.17 ± 0.31	0.34 ± 0.31***	0.66 ± 0.21	0.99 ± 0.28	0.33 ± 0.25***	0.684
all sites	0.98 ± 0.32	1.29 ± 0.40	0.30 ± 0.35***	0.79 ± 0.25	1.13 ± 0.33	0.32 ± 0.30***	0.395

Data are presented as mean ± SD/N

*T0* before periodontally accelerated osteogenic orthodontics surgery, *T1* months after surgery, *CEJ* cemento-enamal junction

^*^P < 0.05; **P < 0.01; ***P < 0.001

At T0, the percentage of gingival thickness ≤ 1 mm at T0 was 65.3%, nearly twice that at the sites of gingival thickness > 1 mm. The percentage of gingival thickness > 1 mm increased to 64.3%. Six months after PAOO surgery, the increase of gingival thickness at ≤ 1 mm sites was significantly higher than at sites > 1 mm (0.36 ± 0.32 mm vs. 0.18 ± 0.31 mm, *P* < 0.001) (Table 3).

**Table 3:** The differences of gingiva thickness increase (mm) before PAOO and 6-month after surgery of gingiva thickness ≤ 1 mm before surgery and gingiva thickness > 1 mm before surgery

	% at T0	% at T1	gingival thickness at T0	gingival thickness at T1	Difference(T1-T0)
≤ 1 mm	65.3	35.70	0.73 ± 0.17	1.08 ± 0.33	0.36 ± 0.32
> 1 mm	34.7	64.30	1.26 ± 0.22	1.44 ± 0.36	0.18 ± 0.31
*P*					< 0.001***

Data are presented as mean ± SD/N

*T0* before periodontally accelerated osteogenic orthodontics surgery, *T1* 6 months after surgery

^***^P < 0.001

### The changes of keratinized gingiva width

The mean keratinized gingiva width at T0 and T1 was 3.88 ± 1.22 mm and 4.92 ± 1.01 mm, respectively, and thus, increased by 1.05 ± 1.24 mm 6 months after PAOO (Table 4, *P* < 0.001). The increase for central incisors, lateral incisors, and canines was 1.03 ± 1.26 mm, 1.12 ± 1.28 mm, and 1.00 ± 1.19 mm, respectively.

**Table 4:** The measurements of keratinized gingiva width before PAOO and 6-month after surgery

Tooth level	T0	T1	Difference(T1-T0)	*P*
central incisors	4.03 ± 1.23 mm	5.06 ± 0.91 mm	1.03 ± 1.26 mm	< 0.001***
lateral incisors	3.95 ± 1.10 mm	5.04 ± 0.94 mm	1.12 ± 1.28 mm	< 0.001***
canines	3.68 ± 1.27 mm	4.69 ± 1.12 mm	1.00 ± 1.19 mm	< 0.001***
all sites	3.88 ± 1.22 mm	4.92 ± 1.01 mm	1.05 ± 1.24 mm	< 0.001***

Data are presented as mean ± SD/N

T0: before periodontally accelerated osteogenic orthodontics surgery, T1: 6 months after surgery

^***^P < 0.001

For the maxilla, the increase for central incisors, lateral incisors, and canines was 0.93 ± 1.25 mm, 1.21 ± 1.64 mm, and 0.78 ± 1.45 mm, respectively. For the mandible, the increase of these different teeth was 1.01 ± 1.26 mm, 1.07 ± 1.10 mm, and 1.15 ± 0.97 mm respectively. However, there was no statistical difference between the maxilla and mandible of keratinized gingiva width increase (Table 5).

**Table 5:** The differences of keratinized gingiva width (mm) before PAOO and 6-month after surgery at different jaw

	maxilla			mandible			*P*
Measurement levels	T0	T1	Difference(T1-T0)	T0	T1	Difference(T1-T0)	
central incisors	4.64 ± 1.03	5.58 ± 0.86	0.93 ± 1.25***	3.63 ± 1.19	4.74 ± 0.78	1.01 ± 1.26***	0.431
lateral incisors	4.40 ± 1.17	5.60 ± 0.92	1.21 ± 1.64***	3.76 ± 1.01	4.80 ± 0.85	1.07 ± 1.10***	0.590
canines	4.44 ± 1.25	5.12 ± 1.08	0.78 ± 1.45***	3.26 ± 1.10	4.41 ± 1.06	1.15 ± 0.97***	0.066
total sited	4.47 ± 1.16	5.41 ± 0.98	0.94 ± 1.43***	3.55 ± 1.12	4.64 ± 0.92	1.11 ± 1.11***	0.184

Data are presented as mean ± SD/N

*T0* before periodontally accelerated osteogenic orthodontics surgery, *T1* 6 months after surgery

^***^P < 0.001

## Discussion

Our preliminary results showed that periodontal soft tissue increases 6-month after PAOO surgery. The gingival thickness increased by 0.30 ± 0.33 mm and the keratinized gingiva width increased by 1.05 ± 1.24 mm. The mean gingival thickness before surgery was 0.91 ± 0.32 mm and was 1.21 ± 0.38 mm at 6 months after PAOO. Furthermore, the sites of gingival thickness of ≤ 1 mm before surgery displayed greater tissue gain than the sites of > 1 mm. Meanwhile, we proposed a digital 3D model based on the combination of digital intraoral scanning and CBCT data to measure gingival thickness with high accuracy and reliability.

Several studies have reported a gain of keratinized gingiva through PAOO, however, few studies analyzed the changes of gingival thickness [5]. Our preliminary results showed that the mean gingival thickness before surgery was 0.91 ± 0.32 mm and 1.21 ± 0.38 mm at 6 months after PAOO, and thus detected a mean gingival thickness gain of 0.30 ± 0.33 mm. To the best of our knowledge, this is the first study that has evaluated the changes of gingival thickness 6 months after PAOO. These results indicated that PAOO could induce increased periodontal soft tissue, including both keratinized gingiva width and gingival thickness.

The 2017 world workshop on the classification of periodontal and peri-implant diseases and conditions reported that it can be assessed by using a periodontal probe to measure the gingival thickness observing the periodontal probe shining and it is assumed the probe will be visible when gingival thickness is thin (≤ 1 mm) and not visible in a thick gingival thickness (> 1 mm) [10]. According to the above criteria, our study showed that the percentage of the gingival thickness ≤ 1 mm was high at 65.3% at T0, while 6 months after PAOO, 64.30% of the sites increased to > 1 mm, which indicated a great improvement of the gingival thickness. Interestingly, the increase of gingival thickness for sites ≤ 1 mm at T0 was significantly higher than sites > 1 mm (0.36 ± 0.32 mm vs. 0.18 ± 0.31 mm, *p* < 0.001). We also found that the gingival thickness increase of the mandible was greater than that of the maxilla at 1 mm, 2 and 3 mm apical to CEJ levels. This result may be due to the gingival thickness of the mandible, whereby all the measurement sites < 1 mm at T0 were obviously thinner than of the maxilla. These same trends appear to indicate that the thinner the gingiva at T0, the greater the increase induced by PAOO surgery, which emphasized the necessarily and effectiveness of the surgery particularly for a thin biotype. The tendency of the increase of gingival thickness and the keratinized gingiva width was in agreement with other previous studies [8]. The current findings all verified the effect of periodontal soft tissue increase including both the thickness and width of gingiva through PAOO.

PAOO surgery was necessary and effective for skeletal Angle Class III malocclusion patients with insufficient quantity of periodontal soft and hard tissues [2], because the risks of further breakdown of periodontal tissues during orthodontic treatment remain high [11, 12]. Several studies have reported that augmented corticotomy increased alveolar bone thickness [13, 14] and horizontal ridge thickness [15]. Moreover, our previous study found a mean gain of 0.65 mm in the labial bone thickness [3]. Together with previous studies, it could be concluded that PAOO surgery could achieve an increase in both periodontal soft and hard tissues increase in skeletal Angle Class III malocclusion patients.

Different invasive and non-invasive methods have been reported to measure soft tissue thickness, including the visual assessment of probe transparency, CBCT use, and horizontal transmucosal bone sounding [16, 17]. While each of these showed some advantages, there is a lack of consensus regarding a reliable, reproducible, and non-invasive approach to precisely assess gingival thickness in clinical practice and research. Digital workflows offer the possibility for precise assessment of the soft tissue thickness by combining intraoral scanning files which could represent details of contour surfaces with CBCT files which could represent details of alveolar bone in three dimensions. Digital assessment of the dimensional features of periodontal and peri-implant soft tissues has been proven a highly reliable and reproducible approach [18, 19]. Recently, study has showed that digital measurement of gingival thickness is comparable with direct clinical assessments performed with transgingival horizontal probing using an endodontic spreader [20]. The high ICC of 0.897 between different examiners in the present study also proved the accuracy and reliability of the digital measurements. A digital approach could be widely used in clinical practice for its non-tissue invasive, reproducible, and reliable nature.

Image registration accuracy of intraoral scanning and CBCT images is the key point in gingival thickness measurement. We used 3Shape Design Studio software which could provide both automatic registration algorithms and manual registration to ensure accuracy [19]. The process of image registration depends on the anatomical landmarks of the different images, such that the corresponding tooth surfaces should be absolutely the same in intraoral scanning and CBCT images [21]. The accuracy and reliability of the combination of digital intraoral scanning and CBCT in our study were proved by the high consistency of two independent examiners. Merging different imaging modalities such as magnetic resonance imaging, multi-detector computed tomography and positron emission tomography to display both osseous and soft tissues has been introduced, including in computer-aided robotic orthopedic surgeries, radiotherapy, and neurosurgery [22]. Digital assessment of the dimensional features of oral soft tissues and osseous tissue has been proven a highly reliable and reproducible approach. In our study, on combining digital intraoral scanning and CBCT, it could provide a distinct border between the gingiva and attached alveolar bone on the registration model, which ensures the accuracy of the gingival thickness measurement. Emilio et al. have reported that digital measurement of gingival thickness using STL-DICOM file superimposition represents a reproducible and reliable method that is comparable with direct transmucosal probing measurements performed with an endodontic spreader [20]. These findings strongly recommended that measurement based on a digital method can be used in clinical practice as a non- invasive, reproducible, and reliable strategy.

The theoretical basis for PAOO was the regional accelerating phenomenon (RAP) [23]. RAP is a natural reaction of the soft and hard tissues, which increases the healing capacities of the affected tissues and is the main biological mechanism of the acceleration of orthodontic tooth movement. RAP accelerated the normal regional healing processes by transient bursts of hard tissue remodeling [24]. For periodontal soft tissue wound healing and regeneration, the major physiological processes of wound-healing are proliferation, protein production and secretion, viability, migration, gene expression, and differentiation, and the healing process involves several different types of cell and numerous growth and differentiation factors [25]. Other studies have reported similar results that growth factors including transforming growth factor-β and basic fibroblast growth factor could regulate the growth and cytodifferentiation of periodontal tissue cells [26–28]. Therefore, soft tissue remodeling and increase might be explained by the accelerated proliferation, migration ability and gene expression induced by growth factors during wound healing process. Furthermore, in vitro laboratory studies and animal models will be needed to verify the above hypothesis in our future work.

## Conclusion

PAOO could improve insufficient periodontal soft tissue in patients with skeletal Angle Class III malocclusion, including the gingival thickness and keratinized gingiva width. The digital 3D model based on the combination of digital intraoral scanning and CBCT data could provide a new digital measurement of gingiva thickness with high accuracy and reliability.

## Supplementary Information

**Additional file 1:** **Figure S1.** Surgical procedurefor augmented corticotomy in a subject with severe skeletal Angle Class IIImalocclusion: **A**) local anesthesia; **B**) full-Thickness flaps were elevatedon the labial aspect, and areas of fenestration and dehiscene were detected; **C**) vertical alveolar decortication wasperformed in the interradicular space; **D**)bone derivative material was placed; **E**)bioabsorbable collagen membrane was placed to completely cover the graft site; **F**) flaps were coronally repositionedand sutured in place.

## Data Availability

The datasets used or analyzed during the study are available from the corresponding author upon reasonable request.
