# Supplementary figures and images for: Periodontal soft tissue increase induced by periodontally accelerated osteogenic orthodontics surgery
Source: BMC Oral Health. 2022 Nov 16;22:506. doi: 10.1186/s12903-022-02566-8 (PMC9670531; doi:10.1186/s12903-022-02566-8)

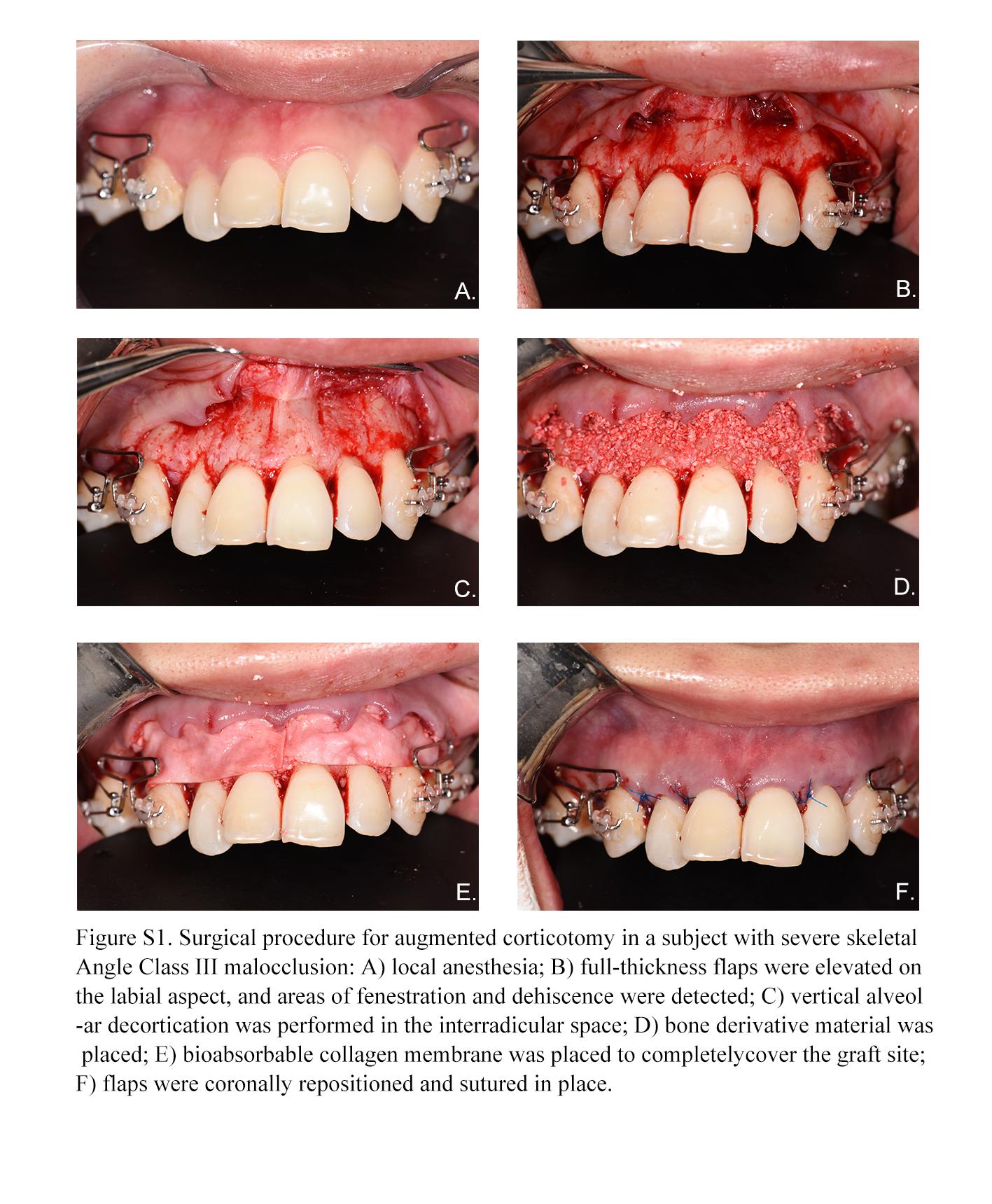

Supplement: Supplementary file 1 — Additional file 1: Figure S1. Surgical procedurefor augmented corticotomy in a subject with severe skeletal Angle Class IIImalocclusion: A) local anesthesia; B) full-Thickness flaps were elevatedon the labial aspect, and areas of fenestration and dehiscene were detected; C) vertical alveolar decortication wasperformed in the interradicular space; D)bone derivative material was placed; E)bioabsorbable collagen membrane was placed to completely cover the graft site; F) flaps were coronally repositionedand sutured in place. [file 12903_2022_2566_MOESM1_ESM.jpg]
